# Deep-Reinforcement Learning-Based Co-Evolution in a Predator–Prey System

**DOI:** 10.3390/e21080773

**Published:** 2019-08-08

**Authors:** Xueting Wang, Jun Cheng, Lei Wang

**Affiliations:** 1 CAS Key Laboratory of Human-Machine Intelligence-Synergy Systems, Shenzhen Institutes of Advanced Technology, Chinese Academy of Sciences, Shenzhen 518055, China; 2 Department of Mechanical and Automation Engineering, The Chinese University of Hong Kong, Shatin 999077, Hong Kong, China

**Keywords:** co-evolution, population dynamics, Monte Carlo simulation

## Abstract

Understanding or estimating the co-evolution processes is critical in ecology, but very challenging. Traditional methods are difficult to deal with the complex processes of evolution and to predict their consequences on nature. In this paper, we use the deep-reinforcement learning algorithms to endow the organism with learning ability, and simulate their evolution process by using the Monte Carlo simulation algorithm in a large-scale ecosystem. The combination of the two algorithms allows organisms to use experiences to determine their behavior through interaction with that environment, and to pass on experience to their offspring. Our research showed that the predators’ reinforcement learning ability contributed to the stability of the ecosystem and helped predators obtain a more reasonable behavior pattern of coexistence with its prey. The reinforcement learning effect of prey on its own population was not as good as that of predators and increased the risk of extinction of predators. The inconsistent learning periods and speed of prey and predators aggravated that risk. The co-evolution of the two species had resulted in fewer numbers of their populations due to their potentially antagonistic evolutionary networks. If the learnable predators and prey invade an ecosystem at the same time, prey had an advantage. Thus, the proposed model illustrates the influence of learning mechanism on a predator–prey ecosystem and demonstrates the feasibility of predicting the behavior evolution in a predator–prey ecosystem using AI approaches.

## 1. Introduction

In recent years, the environment of our planet has become worse and worse, and the ecosystem is facing a crisis of destruction as a result of climate change. In the long run, it may be more important that the crisis is bound to destroy the stable ecosystems that already exist, with consequences likely to persist for millions of years. However, the adaptation and evolution of organisms to the environment is unpredictable due to the sophisticated changes and the lengthy processes, but fortunately, we can make meaningful estimates of those processes by some means [1,2]. Monte Carlo simulation algorithm is one of the most important method to study the temporal and spatial characteristics of large-scale ecosystem [3,4,5,6,7,8,9,10,11,12,13]. Compared with the traditional population dynamics algorithm, the Monte Carlo simulation in two-dimensional space can reveal more details and spatio-temporal characteristics. However, it is still a challenging problem to use it to understand the learning mechanism and adaptation process of the organisms.

Reinforcement learning is one of the most very popular branch of machine learning algorithms, which allows us to study individual behaviors by using simple rules which perform well in complex environments [14]. Individuals can learn and improve their behaviors based on their experience of interacting with the environment by using the reinforcement learning algorithm, try to understand the importance of world features via rewards they receive after each action, and learn the optimal policy to determine which action should be taken at the moment [14,15,16,17,18,19,20]. The algorithm is characterized as an interaction between a learner and environment providing evaluative feedback [21]. Environment here is often conceptualized as a Markov decision process, which is usually described by a tuple of five elements $\left(S,A,P,R,\gamma \right)$. S is the set of states, A is the set of actions, $P\left(s_{t},a_{t},s_{t+1}\right)=p\left(s_{t+1}|s_{t},a_{t}\right)$ is the probability of transiting from state $s_{t}$ to state $s_{t+1}$ after taking action $a_{t}$, $R\left(s_{t},a_{t}\right)=r\left(s_{t},a_{t}\right)$ is the probability of receiving an immediate reward for taking action $a_{t}$ in state $s_{t}$, and γ∈[0,1] denotes the discount factor. A policy is denoted by π:S→A. The goal for the reinforcement learning is to learn a policy $\pi^{*}$ to maximize the expected total discounted returns as follows,

(1) $$J\left(\pi \right)=E[\sum_{k=t}^{T}\gamma^{k-t}r\left(s_{k},a_{k}\right)].$$

Deep-reinforcement learning enables reinforcement learning to be applied in high-dimensional space, which has recently been successfully applied in several challenging domains, ranging from board games [22,23,24,25,26,27] to games or robots [28,29,30]. The most famous result is AlphaGo, which shows that the deep-reinforcement learning algorithms can solve challenging problems [27]. Deep Q-Learning (DQN) method is one of most popular algorithms in deep-reinforcement learning [14,18,22,23,24,25,26,28,31,32,33], in which individuals aim to learn a policy $\pi^{*}$ to maximize the expected total discounted returns. The Q-function of a given policy π is defined as $Q^{\pi }\left(s,a\right)$. During each learning iteration, the Q-values in model-free reinforcement learning are updated as follows,

(2) $$Q\left(s,a\right)\leftarrow Q\left(s,a\right)+\alpha (r+\gamma \max_{a^{\prime }}Q\left(s^{\prime },a^{\prime }\right)-Q\left(s,a\right)).$$

In Equation (2), α represents the learning rate, γ∈[0,1] denotes the discount factor of reward *r*. In particular, DQN uses a neural network to estimate the action–value function *Q*, which is parameterized by θ. In addition, the *experience replay memory* mechanism is used to record population experiences and pass them on to their offspring. Individuals can store their current time experience in the form of tuples $\left(s,a,s^{\prime },r\right)$. A population can obtain their behavior principles for a period of time through the periodic training in the experiences stored by individuals, and update them over time.

To sum up, the deep-reinforcement learning algorithm can be used to describe the behavior changes of individuals affected by complex factors such as their parents, environment or other factors, and the accumulated behavioral evolution will lead to intergenerational population changes. Therefore, we propose a framework based on deep-reinforcement learning algorithms to endow organisms learning ability and high intelligence, and use Monte Carlo simulation algorithm to simulate the intergenerational evolution in large-scale ecosystems. Our research framework allows organisms to use experiences through the learning mechanisms, determine their behavior through interaction with the environment, and to pass on experience to their offspring. Our goal is to explore the learning effects of self-interest driven organisms on population and environmental changes. In our model, predators and prey periodically updated their strategies according to the reception results from their behaviors, which led to changes in population sizes, distributions, and so on. Unlike traditional deterministic strategies, species behavior was influenced by their parental experiences. When organisms faced behavioral choices with similar benefits, they would like to choose the one more frequently used by their parents. In the process of population evolution, some individuals who adopted erroneous behavior may be eliminated by natural selection. This suggested that nature was also involved in shaping those evolution behaviors. In summary, our model used a spatially extended population dynamics models to demonstrate the shaping of nature on species. Meanwhile, the deep-reinforcement learning algorithm allowed the relevant species to co-evolve driven by their own interests. In addition, we studied the risk of extinction caused by the inconsistent learning period and learning speed of predators and prey.

## 2. Model

### 2.1. Monte Carlo Simulation Model

We considered an individual-based predator–prey dynamic ecosystem that performed on a square lattice of linear size *L* with periodic spatial boundary conditions [3,4,8,9,13]. Each site can be either covered by a predator (X), a prey (Y) or empty (∅). The adopted neighborhood was the Moore type, which included eight nearest neighbors. At the beginning of the model, a certain number of individuals were randomly placed on the model. At each time step, a site and one of its Moore neighbor sites were paired chosen at random. We constructed the interactions between individuals and their neighbors based on simplest nature rules as follows,

(3) $$\left\{\begin{array}{c} X+\emptyset \to \emptyset +X\\ X+Y\overset{b_{X}}{\to }X+X\\ X+Y\overset{1-b_{X}}{\to }\emptyset +X\\ Y+\emptyset \overset{b_{Y}}{\to }Y+Y\\ Y+\emptyset \overset{1-b_{Y}}{\to }\emptyset +Y \end{array}\right.$$

In Equation (3), if the chosen two sites were a predator and an empty site, the predator moved to the empty site; If a predator caught and ate a prey, it can reproduce an offspring with reproduction rate $b_{X}$ in the captured prey site. If the reproduction process failed, the predator moved to the captured prey site(the site was empty now); A prey can reproduce an offspring in an empty site with reproduction rate $b_{Y}$. If the reproduction process failed, the prey moved to the empty site. Each predator had an intrinsic counter $f_{X}$ to record the food reserves, and the food reserves reduced over time based on the metabolic rate $1/f_{X}$ and recovered when the predator ate a prey. If a predator did not have enough food in reserve, it starved to die. In order to measure time, we used Monte Carlo Steps (MCS). Usually, one MCS was defined as L×L trials. The Monte Carlo simulation was updated asynchronously, i.e., the interactions changed the sites in real time.

### 2.2. Learning Model

We constructed the Markov Decision Processes (MDPS) defined as the tuple $\left(S,A,T,R,\gamma \right)$ in the Monte Carlo simulation, where S was a finite set of states, A was a finite set of actions, T:S×A×S→R was a function representing the transition probability, R:S×A→R was the reward function and γ∈[0,1] was the discount factor. Let E represented the current environment state. An individual *i* got its observation $o_{i}=E\left(x_{i},y_{i}\right){|}_{v_{r}\times v_{r}}$ based on its coordinate $\left(x_{i},y_{i}\right)$ and its visible range $v_{r}\times v_{r}$. Here, we set $s_{i}\equiv o_{i}$. Then actions A were simplified into the following nine categories: {up,down,left,right,up−left,up−right,down−left,down−right,stand−still}, and $T_{s\to s^{\prime }}^{a}=p\left(s^{\prime }|s,a\right)$ represented the probability of transiting from *s* to $s^{\prime }$ by taking action *a*. As shown in Equation (3), the state–action pair ${\left(s,a\right)|}_{b}$ denoted the actions involving the reproductive process (a predator moved to the site with a prey, or a prey moved to an empty site), where *b* was the reproductive rate ($b_{X}$ for predators and $b_{Y}$ for prey). The state–action pair ${\left(s,a\right)|}_{F}$ denoted the failed actions (a predator moved to a site with a predator, or a prey moved to a site with an individual). Thus, we got,

(4) $$\left\{\begin{array}{cc} p(s|s,a)=1, & {if\left(s,a\right)|}_{F}\\ p(s^{\prime }|s,a)=b, & {if\left(s,a\right)|}_{b}\\ p(s^{\prime \prime }|s,a)=1-b, & {if\left(s,a\right)|}_{b}\\ p(s^{\prime \prime \prime }|s,a)=1, & else. \end{array}\right.$$

$R_{s}^{a}=r\left(s,a\right)$ represented an immediate reward achieved at each state–action pair. π(s):S→A denoted a policy that mapped a state $s_{i}$ to an action π(i)∈A ($\pi^{X}$ for predators, $\pi^{Y}$ for prey). The goals for species were to find optimal policies $\pi^{X*}$ and $\pi^{Y*}$ to maximize the expected total discounted reward as follows,

(5) $$R_{t}=E\left[\sum_{k=t}^{T}\gamma^{k-t}r\left(s_{k},a_{k}\right)\right].$$

where *t* was the current time step and *T* was the total time to run the system. Predators and prey got their population rewards $R^{X}$ or $R^{Y}$ respectively. Then we can write the Bellman equation for the value function of a policy π:

(6) $$V^{\pi }\left(s\right)=r\left(s,\pi \left(s\right)\right)+\gamma \sum_{s^{\prime }\in S}p\left(s^{\prime }|s,\pi \left(s\right)\right)V^{\pi }\left(s^{\prime }\right),$$

and the state–action–value function $Q^{\pi }$:

(7) $$Q^{\pi }\left(s,a\right)=r\left(s,a\right)+\gamma \sum_{s^{\prime }\in S}p\left(s^{\prime }|s,a\right)V^{\pi }\left(s^{\prime }\right).$$

By using Bellman equation as an iterative update, we got

(8) $$Q_{i+1}\left(s,a\right)=E\left[r\left(s,a\right)+\gamma \max_{a^{\prime }}\sum_{s^{\prime }\in S}p\left(s^{\prime }|s,a\right)Q_{i}\left(s^{\prime },a^{\prime }\right)\right].$$

We referred to a neural network function approximation with weights θ as a Q-network to estimate the action–value function, $Q\left(s,a;\theta \right)\approx Q^{*}\left(s,a\right)$. A Q-network can be trained by minimizing a sequence of loss functions $L_{i}\left(\theta_{i}\right)$ that changes at each iteration *i*,

(9) $$L_{i}\left(\theta_{i}\right)=E\left[\sqrt{1+{\left(y_{i}-Q\left(s,a;\theta_{i}\right)\right)}^{2}}-1\right],$$

where $y_{i}=E\left[r+\gamma \max_{a^{\prime }}\sum_{s^{\prime }\in S}p\left(s^{\prime }|s,a\right)Q\left(s^{\prime },a^{\prime };\theta_{i-1}\right)\right]$ was the target for iteration *i*.

In our model, species trained their $Q^{X}$-networks($Q^{Y}$-networks) based on the policy $\pi^{X}$($\pi^{Y}$) to minimize the loss function $L^{X}$($L^{Y}$). We had two population memory buffers $D^{X}$ and $D^{Y}$, to record $\left(s,a,r,s^{\prime }\right)$ of predators and prey at corresponding time steps respectively. In practice, our algorithm only stored the last N experience tuples in the replay memory, and sampled uniformly at random from D when performing updates. We periodically trained the two Q-networks based on the memory buffers, respectively. The training periods of predators and prey were $t_{X}$ and $t_{Y}$.

## 3. Experiments

### 3.1. Reward Functions

We used the Chebyshev distance to represent the distance between two agents. If the chosen two agents had coordinates $\left(x_{1},y_{1}\right)$ and $\left(x_{2},y_{2}\right)$, their Chebyshev distance was,

(10) $$D_{c}=\max(|x_{2}-x_{1}\left|,\right|y_{2}-y_{1}\left|\right).$$

Let $n_{k}\left(Y\right)$ denote the number of preys with a Chebyshev distance of $k=D_{c}$ from the chosen predator. We set the state reward $R^{X}\left(s\right)$ for predators as follows,

(11) $$R^{X}\left(s\right)=\left\{\begin{array}{c} 20n_{1}\left(Y\right)+10n_{2}\left(Y\right)+\sum_{j=3}^{v_{r}}n_{j}\left(Y\right),if\;\sum_{k=1}^{v_{r}}n_{k}\left(Y\right)\neq 0,\\ -10,if\sum_{k=1}^{v_{r}}n_{k}\left(Y\right)=0. \end{array}\right.$$

The reward encouraged predators to eat their nearby prey. On the contrary, the training for prey encouraged prey to move to the direction with fewer predators. In previous study [4], we knew that prey received the most influence from its nearest neighbors. Thus, let $n_{k}\left(X\right)$ denote the number of predators with a Chebyshev distance of $k=D_{c}$ from the chosen prey. We set the state reward $R^{Y}\left(s\right)$ as follows:

(12) $$R^{Y}\left(s\right)=\left\{\begin{array}{c} -10n_{1}\left(X\right)-n_{2}\left(X\right),if\;\sum_{k=1}^{v_{r}}n_{k}\left(Y\right)\neq 0,\\ 10,if\sum_{k=1}^{v_{r}}n_{k}\left(Y\right)=0. \end{array}\right.$$

### 3.2. Data and Architecture of Q-Networks

**Figure 1:**
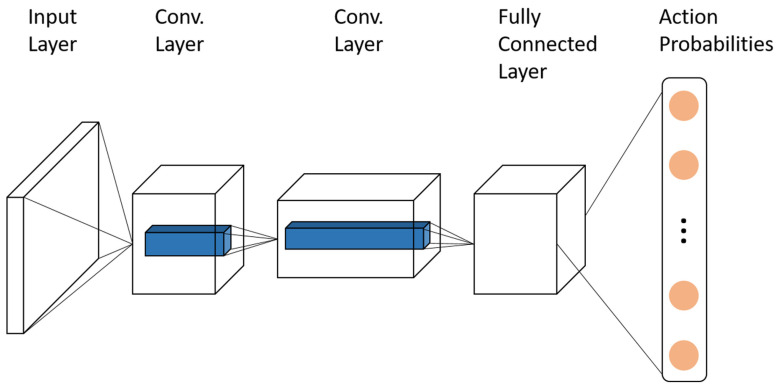
The architecture of our Q-network. The input layer was the observation of the chosen individual, the output layer was Q-values for each action.

All networks were trained by DQN algorithm [22,23], the architecture of the Q-network is shown in Figure 1. Each individual *i* can only know the site states within its range of vision $v_{r}=9$, the center of which was the chosen one. Therefore, the input of the neural network was a 9×9 image produced by the chosen individual. The first hidden layer convolved 32 1×1 filters with stride 1. The second hidden layer convolved 64 1×1 filters with stride 1. The next layer was a fully connected and consisted of 32 rectifier units. All layers included ReLU and batch normalization. The output layer was a fully connected tanh layer with a set of Q-values, one for each action. In the experiments, we used the Adam algorithm with minibatches of size 32. The behavior policy during training was ϵ−greedy with ϵ annealed linearly from 1 to 0.05, and fixed at 0.05 thereafter. The discount factor was set to γ=0.95. The network was trained every MCS. The learning rate was 0.01. The training periods of predators and prey were $t_{X}=t_{Y}=50$.

## 4. Results

### 4.1. Basic Results

We studied the population dynamics with predators and prey on a two-dimension lattice with size L=100. Initially, 500 predators and 2000 prey were randomly placed on the lattice. All simulations ran with initial random spatial distribution. The predator food reserve was set to f=1 initially and decayed by $1/f_{X}=0.1$ at each MCS. From the previous research, we knew that the model can be quickly stabilized without considering individual reinforcement learning. The larger *L* was, the more stable the ecosystem was. For the sake of training time, we only considered the case of L=100. Full exploration of the parameter space was possible only if there were not too many parameters. As in nearly all theoretical models, we did not try to reproduce faithfully a given ecosystem. Our previous research had shown more about the impact of parameters on the ecosystem [4]. Then, we set the max survival age of prey at 100 and the reproduction rate at $\left(b_{X},b_{Y}\right)=\left(0.8,0.1\right)$. The results reported below coming from single runs.

**Figure 2:**
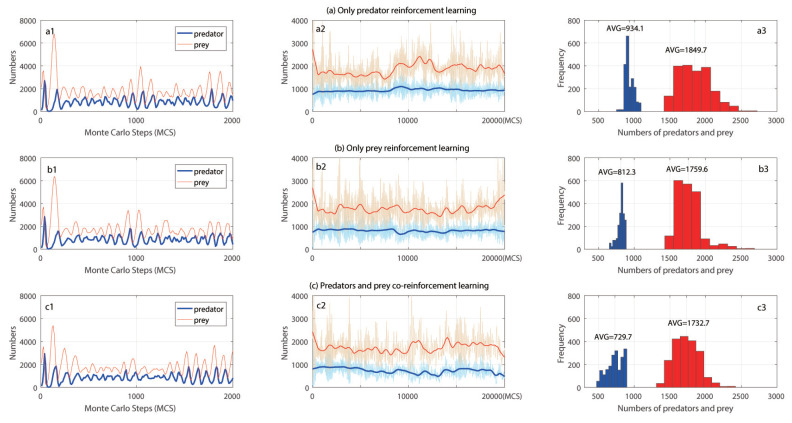
(**a**) The case of only predator reinforcement learning. (**a1**) Numbers of predators and prey evolving through time. From top to bottom, red line represented numbers of prey, blue line represented numbers of predators; (**a2**) The trend of predators and prey evolving through time at 0–20,000 MCS (LOWESS smoother on the numbers); (**a3**) The average numbers of predators and prey and the statistical results of quantitative distributions, left blue one was predators with average number 934.1, red one was prey with average number 1849.7. (**b**) The case that only prey reinforcement learning. (**b1**) Numbers of predators and prey evolving through time. From top to bottom, red line represented numbers of prey, blue line represented numbers of predators; (**b2**) The trend of predators and prey evolving through time at 0–20,000 MCS (LOWESS smoother on the numbers; (**b3**) The average numbers of predators and prey and the statistical results of quantitative distributions, left blue one was predators with average number 812.3, red one was prey with average number 1759.6. (**c**) Co-evolution of predators and prey. (**c1**) Numbers of predators and prey evolving through time. From top to bottom, red line represents numbers of prey, blue line represents numbers of predators; (**c2**) The trend of predators and prey evolving through time at 0–20,000 MCS (LOWESS smoother on the numbers); (**c3**) The average numbers of predators and prey and the statistical results of quantitative distributions, left blue one was predators with average number 729.7, red one was prey with average number 1732.7.

In Figure 2, we considered three different scenarios: only predators reinforcement learning, only prey reinforcement learning, and predator–prey co-learning. Similar to previous studies [13], there was an oscillating relationship between predators and prey. As the number of predators grew, more prey would be captured, and prey population shrunk. Due to the lack of food, the reduction in the number of preys would lead to a reduction number of predators over a period of time. Then, with the decreasing numbers of predators, the population of prey started to grow.

To investigate the trend of predators and prey through 20,000 MCS, we carried out LOWESS smoother on the curve of numbers of predator and prey. Due to the slow decline in the exploration rate, the evolution of the deep-reinforcement learning network had a relatively stable impact on the system. In Figure 2(a2), the predator had a small increase at about 8000 MCS, and the prey oscillated obviously. After the number of preys declined at around 8000 MCS, the prey population showed a slow upward trend. This suggested that the learning predators find a better strategy to hunt prey than the random strategy, and meanwhile numbers of predators and prey both increased. In Figure 2(b2), the prey oscillated less than that in Figure 2(a2), and at the end of the trial, the prey population increased. At around 9000 MCS, the number of predators decreased.

When the predators and prey co-evolved, predators and predators updated their networks according to each other’s behavior, which significantly reduced the oscillations of the ecosystem. The two networks had potential antagonistic factors on the ecosystem and interacted with each other. As a result, the quantity of the two populations decreased after 15,000 MCS. We can also know that the number of predators was the largest when only the predators learned. This was because the prey in the ecosystem always used the randomly strategies, which was regular and learnable for the evolving predators. In previous studies [4], the learning and evolution of predators had led to more prey being hunted, thus reducing the numbers of predators and prey. Our results showed a smarter self-evolution of predators. When prey learned alone, we found that both predator and prey populations were lower than the case that only predators learned. The increasing number of preys allowed predators to have plenty of food. Although prey learned better behaviors, it was difficult for prey to increase the number of their population. The average numbers of predators and prey were the lowest in the case of co-evolution, which made it difficult for the two population to find the most appropriate strategy to interact with others. When predator and prey co-evolved, their networks were potentially adversarial, which may be a factor in reducing the numbers of the two species.

### 4.2. Applications of the Networks

**Figure 3:**
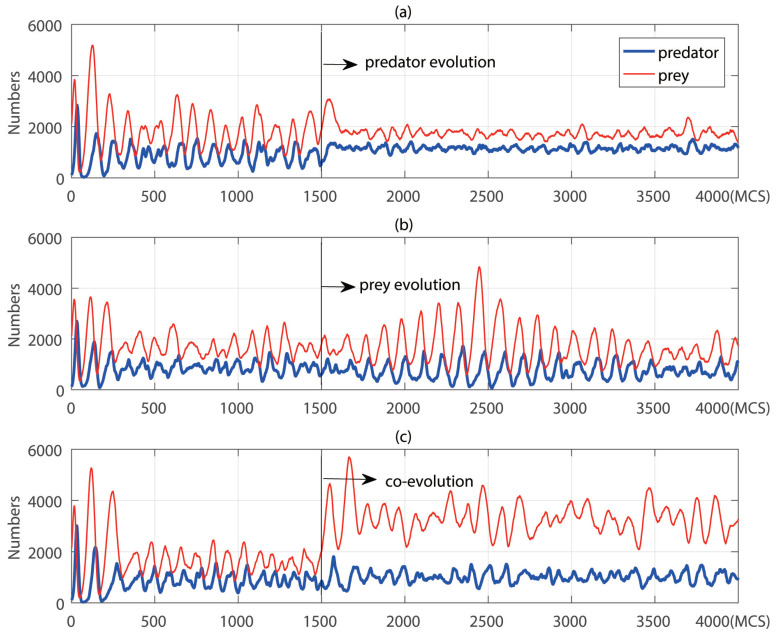
Numbers of predators and prey evolving through time. The first 1500 MCS showed the basic ecosystems, in which species acted randomly. (**a**) The case that only predators evolved, the trained network from Figure 2a; (**b**) The case that only prey evolved, the trained network from Figure 2b; (**c**) The case that predators and prey co-evolved, the trained network from Figure 2c.

In the section, we studied the application of the predator and prey networks on an original ecosystem. In the previous section, we had trained three kinds of networks, which were the reinforcement learning of predators, the reinforcement learning of prey and the reinforcement learning of both predators and prey. We applied those networks on the normal simulation ecosystem. As shown in Figure 3, in the former 1500 MCS of the simulation, both predators and prey took random actions. The exploration rate was fixed to 0.05 and parameters of the network would not update through time. Figure 3a showed the evolution of predators, the predators took their actions using the deep-reinforcement learning network trained in Figure 2a after 1500 MCS. It can be seen from Figure 3a that after predators used a learning strategy, the numbers of predators and prey increased and became stable. We can go further to say that the learning of predators played a positive role in the construction and stability of ecosystems.

Only prey learned their behaviors using deep-reinforcement learning in Figure 3b after 1500 MCS. It can be seen that the oscillations brought periodic changes in the number of preys. However, the short-term growth of prey led to a rapid increase in the number of predators, i.e., reinforcement learning cannot enable prey to obtain good measures to deal with the increasing predators. After oscillations, predators and prey can adapt to the ecosystem again, and the ecosystem became stable. However, during the oscillations brought by prey’s reinforcement learning, the number of predators oscillated periodically to a low level, which made predators face a higher risk of extinction. In Figure 3c, predators and prey both used the deep-reinforcement learning strategies after 1500 MCS. When co-evolutionary networks were added to the ecosystem, the number of predators and prey increased significantly, although the co-evolution network did not perform well in the evolutionary process as shown in Figure 2. This suggested that co-evolution a significant impact on species if the learning took place rapidly instead of slowly. We can go further to say that in an evolutionary arms race between predators and prey, prey gained more numbers and predators maintained the stability of the ecosystem.

### 4.3. Inconsistent Co-Evolution of Species

Here, we examined the inconsistent learning periods and speed of predators and prey. We considered four cases and simulated 20,000 MCS, respectively. The four inconsistent cases are shown in Table 1: case4 represented the learning of predators began when the ecosystem started to run, and prey began to learn at 500 MCS; case5 represented the learning of prey began when the ecosystem started to run, and predator began to learn at 500 MCS; case6 represented the training period for predators was 50 MCS and the training period for prey was 100 MCS; case7 represented the training period for predators was 100 MCS and the training period for prey was 50 MCS. The results of mean numbers of predators and prey on the four cases and the basic four cases in Figure 2 are shown in Table 1. Notably, case0 showed the basic line of the model where both predators and prey do not learn.

**Figure 4:**
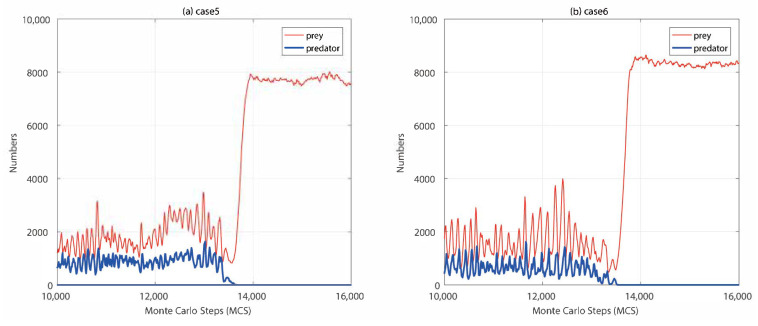
Numbers of predators and prey evolving through time. (**a**) Prey began to learn before predators 500 MCS (case5 in Table 1). (**b**) The training period of predators was 50 MCS, the training period of prey was 100 MCS (case6 in Table 1).

**Figure 5:**
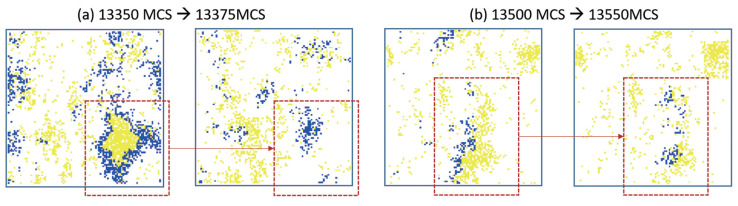
The spontaneous spatial patterns in case5 that prey began to learn before predators 500 MCS (case5 in Table 1). (**a**) showed the distributions on 13,350 MCS and 13,375 MCS. (**b**) showed the distributions on 13,500 MCS and 13,550 MCS.

As can be seen from Table 1, the maximum average number of predators was obtained when predators learned alone(case1), which suggested that the learning of predators had a positive impact on both their population and prey population. The learning of prey in case2 made the number of their population higher than the base line case0, which suggested that the learning prey was also effective. However, compared with case1, the learning of prey had less positive effect on its population. The co-evolution of predators and prey had similar problems. The learning of predators and prey interfered with each other, resulting in a decline in the number of predators, and the increase in the number of preys was not as large as that in the case of predators learning alone. When predators and prey can coexist, prey achieved a highest number of population where prey learned 500 MCS later than predators. In our model, the learning of prey put the ecosystem at risk of collapse and the immediate consequence of prey’s reinforcement learning was the extinction of predators. It can be found from the results that the predator became extinct in case5 and case6. The two examples showed that predators faced a greater risk of extinction in the process of inconsistent co-evolution. If predators learned after their prey, they may become extinct because they lag behind the evolution of their prey. If predators learned faster than prey, the predator’s strategy training would be completed before their prey. Then prey learned against the predator’s trained strategy leading to a high risk of predator extinction, as shown in Figure 4. Figure 5 showed the spontaneous spatial patterns when the evolution proceeded. The predators surrounded and hunted their prey, as shown in Figure 5a. The predators can pursuit their prey in Figure 5b, and then the predators were divided into two groups to pursue their prey.

## 5. Conclusions

In this paper, we have presented a deep-reinforcement learning architecture for species evolving in a large-scale ecosystem. In our model, deep-reinforcement learning was used to represent the adaptation and evolution of predators and prey to the ecosystem. Unlike previous ecosystem simulations, the learning of species was characterized by a steady increase in the automation of traditionally human-based decision processes. We focused on the reinforcement learning effects of both predators and prey. Our results showed that predators had an essential influence on ecosystem structuring and stability through their learning. As the top-level creature in the food chain, predators can learn to obtain appropriate hunting strategies and increase their population along with their prey (Figure 2). Owing to easily obtain the behavior pattern of the unlearned prey, the predators can learn a more reasonable adaptation and evolution mode. Moreover, the learning predators had the opportunity to form spontaneous collaborations to surround and hunt their prey, as well as to pursue their prey in groups (Figure 5). However, co-evolution of the two species had resulted in fewer predators and prey due to their potentially antagonistic evolutionary networks. The learning effect of prey on its own population was not as good as that of predators. Meanwhile, the learning of prey increased the risk of extinction of predators, and the inconsistent learning periods and speed of prey and predators aggravated this phenomenon (Figure 4).

In response to the risk of extinction, predators need to adjust their learning speed to prevent their evolution falling behind prey. Furthermore, predators’ learning stopped earlier than their prey may lead to extinction of their population. Therefore, we believed that the reinforcement learning of predators had an important and positive impact on the ecosystem, especially in terms of population size and biodiversity. In addition, we considered the direct application of the above learned strategies on stable ecosystems (Figure 3). Similar with previous conclusion, the direct application of predators’ learning strategies made the ecosystem more stable, and both the two species had seen an increase in their populations. The application of prey’s learned strategies made the ecosystem oscillate, and the wild oscillations in prey exposed predators to the risk of extinction. From the application results of co-evolution, the number of preys had increased significantly. This suggested that if the learnable predators and prey invade an ecosystem at the same time, prey may have an advantage. To sum up, deep-reinforcement learning enabled individuals to seek a better way to survival of their population in the traditional simulation model from the point of view of self-interests. We can go further to say that the process of learning and adaptation of predators was one of the important factors in maintaining ecosystem stability and protecting biodiversity.

## Figures and Tables

**Table 1:** Some statistics of predators and prey in different cases.

	Predator			Prey			Predator	Prey
	Evolution	Start Time	Period	Evolution	Start Time	Period	Mean	Mean
case0	no	-	-	no	-	-	881.28	1654.56
case1	yes	0	50	no	-	-	934.49	1849.44
case2	no	-	-	yes	0	50	812.71	1759.75
case3	yes	0	50	yes	0	50	729.90	1732.24
case4	yes	0	50	yes	500	50	824.87	1944.30
case5	yes	500	50	yes	0	50	565.16	3617.24
case6	yes	0	50	yes	0	100	532.24	3790.19
case7	yes	0	100	yes	0	50	819.26	1922.06
